# Posttraumatic midazolam administration does not influence brain damage after experimental traumatic brain injury

**DOI:** 10.1186/s12871-022-01592-x

**Published:** 2022-03-04

**Authors:** Anne Sebastiani, Simone Bender, Michael K. E. Schäfer, Serge C. Thal

**Affiliations:** 1 Department of Anesthesiology, HELIOS University Hospital Wuppertal, University of Witten/Herdecke, Heusnerstrasse 40, 42283 Wuppertal, Germany; 2 Department of Anesthesiology, University Medical Center of the Johannes Gutenberg University, Mainz, Germany

**Keywords:** Midazolam, Flumazenil, Benzodiazepines, γ-aminobutyric acid-A receptor, Neurotoxicity, Traumatic brain injury, Controlled cortical impact, Anesthesia, Sedation, Neurological function

## Abstract

**Background:**

The benzodiazepine midazolam is a γ-aminobutyric acid (GABA)-A receptor agonist frequently used for sedation or stress control in patients suffering from traumatic brain injury (TBI). However, experimental studies on benzodiazepines have reported divergent results, raising concerns about its widespread use in patients. Some studies indicate that benzodiazepine-mediated potentiation of GABAergic neurotransmission is detrimental in brain-injured animals. However, other experimental investigations demonstrate neuroprotective effects, especially in pretreatment paradigms. This study investigated whether single-bolus midazolam administration influences secondary brain damage post-TBI.

**Methods:**

Two different midazolam dosages (0.5 and 5 mg/kg BW), a combination of midazolam and its competitive antagonist flumazenil, or vehicle solution (NaCl 0.9%) was injected intravenously to mice 24 h after experimental TBI induced by controlled cortical impact. Mice were evaluated for neurological and motor deficits using a 15-point neuroscore and the rotarod test. Histopathological brain damage and mRNA expression of inflammatory marker genes were analyzed using quantitative polymerase chain reaction three days after insult.

**Results:**

Histological brain damage was not affected by posttraumatic midazolam administration. Midazolam impaired functional recovery, and this effect could not be counteracted by administering the midazolam antagonist flumazenil. An increase in *IL-1β* mRNA levels due to postinjury application of midazolam was reversible by flumazenil administration. However, other inflammatory parameters were not affected.

**Conclusions:**

This study merely reports minor effects of a postinjury midazolam application. Further studies focusing on a time-dependent analysis of posttraumatic benzodiazepine administration are required.

## Background

Patients suffering from traumatic brain injury (TBI) need sedation to induce anxiolysis, prevent agitation, and allow mechanical intubation [1–3]. Benzodiazepines are the frequently used agents for the sedation of patients with TBI, especially in cases where the standard agent propofol alone does not achieve sufficient sedation depth. These agents generally protect injured brains, especially in comatose patients with severe brain lesions [4], an effect partially attributed to its anticonvulsant properties [5]. Benzodiazepines are nonselective central nervous system depressants that increase chloride ions’ conductance by interacting with a binding site between the α1 and γ2 subunits of γ-aminobutyric acid (GABA)-A receptors. This class of substances reduces cerebral blood flow, cerebral metabolic rate, oxygen consumption, and intracranial pressure and increases seizure threshold [6].

Studies have reported divergent results regarding the disadvantages/advantages of benzodiazepines in the peritraumatic phase. There is extensive evidence indicating that potentiation of GABAergic transmission is detrimental in brain-injured animals [7]. Posttraumatic administration of the GABA-A agonist propofol caused long-term neurotoxic effects that were mediated through the proBDNF-p75 neurotrophin receptor (p75NTR) pathway [8, 9]. Consistent with these findings, postinjury antagonization of the benzodiazepine-binding site on GABA-A receptors with flumazenil improved cognitive function after experimental TBI in immature rats [10]. In contrast, increasing evidence shows that preinjury treatment with benzodiazepines improves outcomes in experimental studies. A single, preinjury dose of diazepam reduced mortality and cognitive impairment after fluid percussion brain injury in rats. However, it was not effective when delayed to 15 min after trauma [11]. These results suggest that the beneficial effects of benzodiazepines depend on the timing of treatment and the disease model.

Midazolam is an inexpensive benzodiazepine that is most suitable for sedation in patients with TBI. Unlike other benzodiazepines, such as lorazepam or diazepam, it has a short, context-sensitive *half-time* with rapid onset and offset of action [12]. Our previous study shows that TBI induces the expression of p75NTR 24 hours after experimental TBI, which is associated with proapoptotic signaling. A superinduction of p75NTR further exacerbated P75NTR-mediated cell death through the inhibition of neurotrophin procession by propofol. We hypothesized that the GABA-ergic stimulation by midazolam enhances brain damage in a similar fashion as propofol. In the present study, we therefore investigated the effect of a delayed single-bolus midazolam application at 24 h after trauma, at the peak of p75NTR expression [13], on histological brain damage, neuroinflammation, and neurological function at 3 days after insult to explore whether posttraumatic midazolam application is neurotoxic in a posttreatment paradigm as previously demonstrated for propofol.

## Methods

### Experimental Animals

This study used 51 adult male C57BL/6 mice (weighing 19.7–26.4 g; Charles River Laboratory, Sulzfeld, Germany). Animal care before and during experiments adhered strictly to the guidelines of the Johannes Gutenberg University, Mainz, Germany. The mice remained in their home cages with constant access to food and water. The Animal Ethics Committee approved all experiments of the Landesuntersuchungsamt Rheinland-Pfalz, Germany (protocol number 23 177-07/ G 12-1-010) in accordance with the ARRIVE guidelines. We made every effort to minimize the number of animals used and their suffering.

### Experimental Groups

**Fig. 1:**
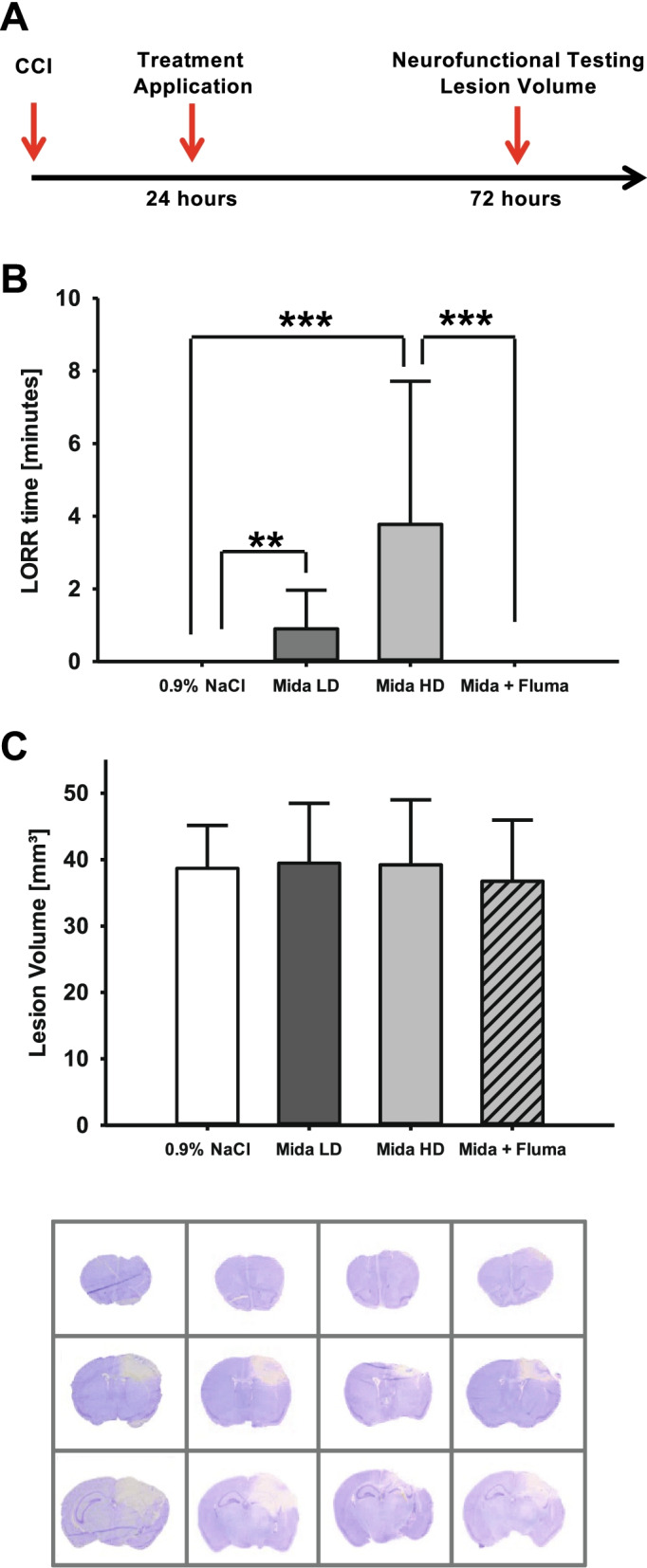
Righting reflexes and brain lesiom volume. **A** Timeline of the study. **B** Loss of righting reflex (LORR) was measured by an investigator blinded to the group allocation. There was a dose-dependent increase in LORR. The vehicle group and the group that received midazolam and flumazenil simultaneously did not show any LORR. **C**, 72 h after injury, the cresyl-violet-stained sections obtained from mice exposed to midazolam or midazolam plus flumazenil application at 24 h after trauma were evaluated for brain damage and compared with the vehicle group (normal saline, NaCl 0.9%). Brain lesion volume was not affected by posttraumatic midazolam or flumazenil administration. Representative cresyl-violet-stained sections at the coronal plane from 1.70 mm anterior to the bregma, 0.46 mm posterior to the bregma, and 1.46 mm posterior to the bregma; *n* = 9 mice for the Mida HD and the Mida HD + Fluma group, *n* = 11 mice for the Mida LD and the vehicle group. *P < 0.05; data are presented as mean ± SD; Mida LD, midazolam low-dose group; Mida HD, midazolam high-dose group; Mida HD + Fluma, midazolam high-dose plus flumazenil.

A total of 51 animals were studied. The group sizes were planned as follows: *n* = 11 animals were planned for the vehicle group, the low-dose midazolam group, the group of animals that received midazolam plus flumazenil low-dose and the group that received high-dose midazolam solution to allow one drop-out per group (Fig. 1A). During sedation, mice were persistently responsive to pain stimuli by pinprick. Due to a randomization error one more animal was included in the high-dose group (*n* = 12). Three mice in the high-dose midazolam group did not lose their righting reflex, whereas one mouse that received midazolam plus flumazenil showed a loss righting reflex, indicating insufficient antagonization of midazolam. Furthermore, one mouse in the midazolam plus flumazenil group was found dead in the cage one day after trauma. These animals were excluded from further analysis.

After exclusion, the final group size was *n* = 11 each for the vehicle and the low-dose midazolam group and n = 9 each for the group of animals that received high-dose midazolam or midazolam plus flumazenil. In addition, one naïve animal group was investigated (*n* = 6).

### Experimental TBI

Animals were placed on a temperature-controlled heating pad to maintain body temperature at 37°C during the surgical procedure. General anesthesia was induced with 4 vol% isoflurane (AbbVie, Wiesbaden, Germany) and maintained with 1.5–2 vol% isoflurane through a face mask. Trauma was induced by controlled cortical impact (CCI) as previously described [14]. Briefly, after craniotomy on the right rostrocaudal plane, a mechanical lesion was induced on the right parieto-temporal cortex using a custom-fabricated impactor (L. Kopacz, Germany) and with the following parameters: tip diameter of 3 mm, 1.5 mm brain penetration, impact duration of 150 ms, and impact velocity of 8 m/s. After trauma, the craniotomy was immediately closed using histoacrylic glue (B Braun Melsungen AG, Melsungen, Germany), and wounds were closed with sutures. Mice were returned to their cages and placed in an incubator (33°C, 35% humidity; IC8000, Draeger, Germany) for 2 h. Animals regained consciousness within 10 min after the induction of trauma.

### Treatment and Drugs

Animals were randomized to four different treatment regimens as follows: midazolam solution (Midazolam hydrochloride, Hameln pharma plus GmbH, Hameln, Germany) was administered at a dose of 0.5 mg/kg body weight (low-dose group, LD) or 5 mg/kg body weight (high-dose group, HD). One group received midazolam 5 mg/kg body weight (high dose) plus flumazenil (Flumazenil-hameln, Hameln pharma plus, Hameln, Germany) 0.5 mg/kg body weight. All drugs were dissolved or diluted in normal saline (0.9% NaCl) and injected at a volume of 10 mL/kg body weight. The vehicle group received 10 mL/kg body weight normal saline (0.9% NaCl). All drugs were administered 24 h after CCI in equal volumes by IV injection into the tail vein by an investigator blinded to the group allocation. A blinded observer scored the mice for loss of righting reflex and measured the time after injection until end of the loss of righting reflex, as described previously [15, 16].

### Motor Coordination

Motor coordination was analyzed by the rotarod test as described previously and by an investigator blinded to the group allocation [17]. After 3 days after TBI, mice were tested twice (two rounds of testing task) before euthanasia. The mean maximum speed and the mean latency time to balance until fall from the rod were recorded using a five-lane accelerating rotarod device (Panlab Rota Rod, Harvard Apparatus, Holliston, MA). The rotarod speed was increased linearly from 4 to 40 rpm over 5 min. The investigation was completed when the mice fell off the rods.

### Neurological Severity Score

An investigator blinded to the group allocation evaluated the mice before and at 3 days after TBI using a modified neurological severity score consisting of 10 different tasks for evaluating motor ability, alertness, balancing, and general behavior [18]. A 15-point scale was used where 0 indicates no neurological impairment and 15 indicates severe neurological dysfunction, as described previously [19].

### Histological Evaluation of Brain Damage

Animals were euthanized under isoflurane anesthesia 72 hours after CCI, after which their brains were carefully removed, snap-frozen in powdered dry ice, and stored at −20°C. Brains were cut in the coronal plane using a cryostat (HM 560 Cryo Star; Thermo Fisher Scientific, Walldorf, Germany). Sections (10 μm in thickness) were collected at 500-μm intervals and stained with cresyl violet. The area of both hemispheres and contused brain tissue, defined as the region lacking cresyl violet staining, was determined. The contusion volume was calculated using a computerized image system (Delta Pix Insight; Delta Pix, Maalov, Denmark) by an investigator blinded to the experimental group randomization. Lesion volumes were calculated by multiplying the lesion areas obtained from 16 consecutive sections with a 500-μm distance interval [0.5 × (A1 + A2+ A3 + … + An)] [20].

### Gene Expression Analysis

Tissue samples of the right upper quadrant (right parieto-temporal cortex) from the brain sections of injured brains were collected during histological processing and snap-frozen in liquid nitrogen as previously described [13]. Samples were stored at −80°C until processing. Quantification of mRNA was performed using a real-time polymerase chain reaction. Absolute copy numbers of target genes were normalized against the housekeeping gene cyclophilin A (PPIA) [21]. Samples were homogenized in QIAzol® reagent (Qiagen). RNA isolation was performed using the RNeasy® Lipid Tissue Mini kit (Qiagen) per the manufacturer’s instructions. Table 1 shows the primer sequences. The same amounts of cDNA were amplified in duplicates using Absolute Fast SYBR Green Mix (Thermo Fisher Scientific) for PPIA and Nos2, Absolute Blue SYBR Green for Tnfa, and Light-Cycler 480 Probes Master (Thermo Fisher Scientific) for Il1b and Il6 according to the manufacturer’s instructions.

**Table 1:** Primers and probes used for real-time polymerase chain reaction

PCR Assay (product length, annealing temperature)	Oligonucleotide Sequence (5′-3′)	Gene Bank Number
Cyclophilin A (PPIA), [212 bp, 58°C]	Forw: 5′-GCGTCTSCTTCGAGCTGTT-3′ Rev: 5′-RAAGTCACCACCCTGGCA-3′ FL: 5′-GCTCTGAGCACTGGRGAGAAAGGA-FL Cy5: Cy5-TTGGCTATAAGGGTTCCTCCTTTCACAG-Phos	NM_008907
TNF-α (Tnfa), [212 bp, 62°C]	Forw: 5′-TCTCATCAGTTCTATGGCCC-3′ Rev: 5′-GGGAGTAGACAAGGTACAAC-3′	NM_ 008361
IL-1β (Il1b), [348 bp, 55°C]	Forw: 5′-59-GTGCTGTCGGACCCATATGAG-3′ Rev: 5′-CAGGAAGACAGGCTTGTGCTC-3′ FL: 5′-TAATGAAAGACGGCACACCCACCC-FL Cy5: Cy5-TTGGCTATAAGGGTTCCTCCTTTCACAG-Phos	NM_008361
IL-6 (Il6), [141 bp, 55°C]	Forw: 5′-GAGGATACCACTCCCAACAGACC-3′ Rev: 5′-AAGTGCATCATCGTTGTTCATACA-3′	NM_031168
iNOS (NOS2), [312 bp, 58°C]	Forw: 5′-TGTGTCAGCCCTCAGAGTAC-3′ Rev: 5′-CACTGACACTYCGCACAA-3′ R640: Red-GCTCCTCCCAGGACCACACCC-Phos FL: 5′-GAAGCCCCGCTACTACTCCATC-FL	NM_010927

*PCR* Polymerase chain reaction, *Forw* Sense primer, *Rev* Antisense primer, *Cy5* Cyanine 5, *Phos* Phosphate, *FL* Fluorescein

### Statistical Analysis

Data were analyzed using the Sigma Plot 13 Statistical Software package (Systat Software, Inc., San José, USA). Exact Wilcoxon–Mann–Whitney tests were performed. Values were adjusted for multiple comparisons using the Holm–Bonferroni method. Data are expressed as mean ± SD. A P level of <0.05 was considered statistically significant. Previously published data from our research group were used to calculate the number of animals. For the primary outcome “lesion volume“ we calculated a group size number of n = 10 when expecting a biological effect by 30%, an α error of 0.05 and a statistical power of 0.9.

## Results

### Low- and High-dose Midazolam Exert a Short-lasting Sedative Effect

The injected doses of midazolam did not induce deep sedation. The animals were tested for loss of righting reflex (LORR) and the duration was recorded. Both dosages of midazolam induced a short-lasting LORR effect, whereas the injection of the vehicle, or the mixture of midazolam and its antagonist flumazenil, had no effect on the LORR (high-dose midazolam: 3.8 ± 3.9 min, low-dose midazolam: 0.8 ± 1.0 min, *P* = 0.007 for low-dose midazolam versus vehicle, *P* < 0.001 for high-dose midazolam versus vehicle group, and *P* < 0.001 for high-dose midazolam versus midazolam plus flumazenil; Fig. 1B).

### A single posttraumatic bolus administration of midazolam does not influence brain lesion volume

For determining the effect of midazolam on brain damage, we quantified the brain lesion volume 72 h after inducing experimental brain trauma. Midazolam administration at 24 h post insult did not influence the increase in lesion volume compared with vehicle treatment. The combination of midazolam and its antagonist flumazenil also had no effect on the lesion size (vehicle: 38.7 ± 6.5 mm^3^; low-dose midazolam: 39.5 ± 9.0 mm^3^; high-dose midazolam: 39.2 ± 9.8 mm^3^; high-dose midazolam + flumazenil: 36.8 ± 9.2 mm^3^, *n* = 9–11 mice/group; Fig. 1C).

### Posttraumatic Midazolam Impairs Neurofunctional Recovery

**Fig. 2:**
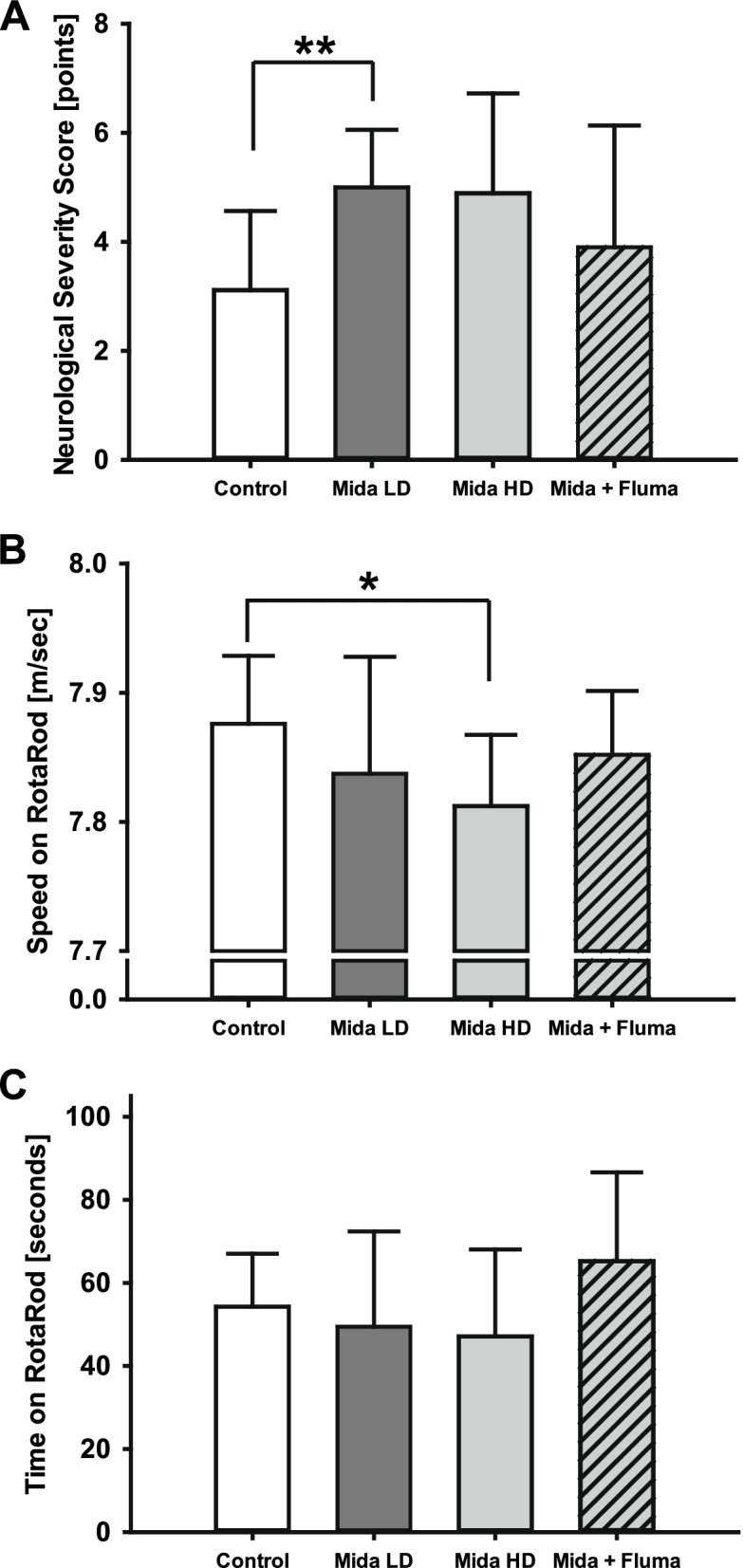
Neurofunctional recovery. **A** Neurological function was examined 3 days after experimental TBI using the neurological severity score (0 point = no impairment, 15 points = maximal impairment). All mice exhibited moderately impaired neurological function after trauma. Mice treated with low-dose midazolam exhibited significantly impaired neurological function compared with mice in the vehicle group. **B** effect of midazolam or midazolam plus flumazenil treatment on motor function was evaluated using the rotarod test. The performance of the high-dose midazolam group was significantly poorer than that of the vehicle group (normal saline, NaCl 0.9%), **P < 0.01, *P < 0.05, *n* = 9 mice for the Mida HD and the Mida HD + Fluma group, *n* = 11 mice for the Mida LD and the vehicle group; data are presented as mean ± SD; Mida LD, midazolam low-dose group; Mida HD, midazolam high-dose group; Mida HD + Fluma, midazolam high-dose plus flumazenil.

To explore whether midazolam administration affects posttraumatic neurofunctional recovery, we determined a 15-point neurological severity score. Three days after insult, all animals exhibited impaired neurological function. Neurological impairment was significantly enhanced by low-dose midazolam administration (5.0 ± 1.1 points, *P* = 0.007 versus vehicle) and trended towards increased neurological deficits in the high-dose midazolam group (4.9 ± 1.8 points, *P* = 0.059 versus vehicle) compared with vehicle-treated mice (3.1 ± 1.5 points). This effect was not influenced by the antagonist flumazenil (3.9 ± 2.2 points, *P* = 0.336 versus high-dose midazolam; *n* = 9–11 mice/group; Fig. 2A).

We also evaluated locomotion and coordination using the rotarod test. Three days after injury, mice treated with high-dose midazolam performed worse on the rotarod task, as determined by maximal walking speed (high-dose midazolam: 7.81 ± 0.06 m/s, vehicle: 7.88 ± 0.05 m/s, *P* = 0.033). Similar to the neuroscore results, this effect remained unaffected by flumazenil administration (7.85 ± 0.05 m/s, *P* = 0.116; Fig. 2B). There were no significant differences between the groups in terms of latency on the rotating rod (Fig. 2C).

### Posttraumatic Midazolam Increases IL-1β mRNA Expression Levels

**Fig. 3:**
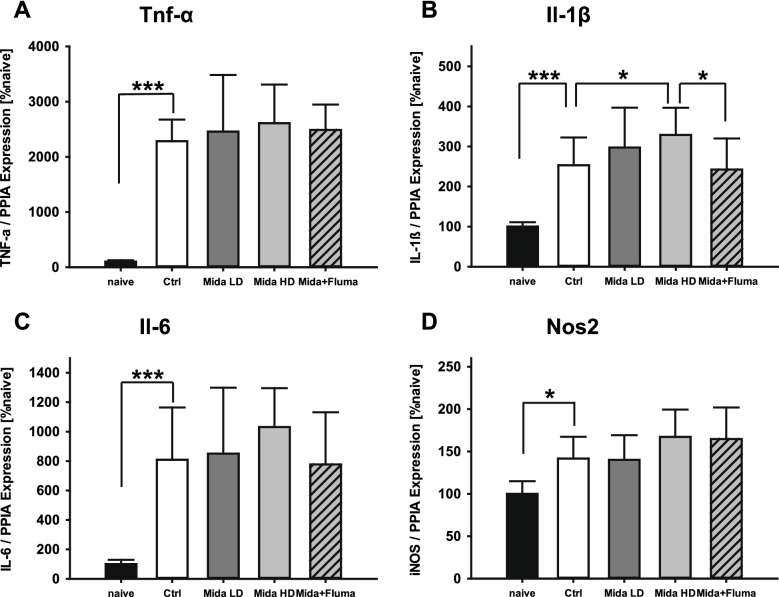
Cerebral inflammation. mRNA expression of the inflammatory markers TNF-α (**A**), IL-1β (**B**), IL-6 (**C**), and iNOS (**D**) relative to peptidylprolyl isomerase A (PPIA) 72 h after injury. Experimental TBI increased the mRNA expression of all proinflammatory markers. IL-1β mRNA expression was increased by high-dose midazolam, which was abrogated by flumazenil; naive: *n* = 6 mice, treatment groups: *n* = 9 mice for the Mida HD and the Mida HD + Fluma group, *n* = 11 mice for the Mida LD and the vehicle group, ***P < 0.001, *P < 0.05; data are presented as mean ± SD; Mida LD, midazolam low-dose group; Mida HD, midazolam high-dose group; Mida + Fluma, midazolam high-dose plus flumazenil.

We quantified the mRNA expression levels of the proinflammatory marker genes *TNF-α*, *IL-6*, *IL-1β*, and *iNOS* in ipsilesional brain tissues (Fig. 3). All animals had increased expression levels three days after CCI. Mice treated with high-dose midazolam had a significantly higher *IL-1β* mRNA expression than those treated with vehicle (high-dose midazolam: 328.7 ± 68.0 %naive; vehicle: 253.3 ± 69.1 %naive; *P* = 0.041, Fig. 3B), abrogated by treatment with the specific antagonist flumazenil (midazolam plus flumazenil: 242.6 ± 77.4 %naive; *P* = 0.028 vs high-dose midazolam). The mRNA expression levels of *TNF-α*, *IL-6*, and *iNOS* were not influenced by midazolam treatment.

## Discussion

Short-acting benzodiazepines such as midazolam are commonly used to manage patients with TBI [22–24]. In this study, we examined the effect of two dosages of a single-bolus administration of midazolam compared with vehicle solution and the combined application of midazolam and its antagonist flumazenil. Although there was no effect on lesion volume by posttraumatic midazolam administration, we observed an aggravation in motor deficits after midazolam administration and increased *IL-1β* mRNA levels significantly compared to the control groups.

Benzodiazepines remain the core medication for sedation in the ICU or surgical procedures. Short-acting agents such as midazolam are frequently used to treat seizures, acute mania, motor agitation, and other psychiatric emergencies in the TBI population. In brain injury, the effects of benzodiazepines are complex. Some clinicians consider benzodiazepines as a group of potentially detrimental medications for patients suffering from stroke or TBI [24–26]. A study investigated the effect of midazolam in patients who had suffered a recent transient cerebral ischemic attack and were neurologically intact. The results indicated the reemergence of previous focal deficits after midazolam administration in a dose that produced mild sedation [27]. The present study could demonstrate an influence on motor deficits measured by the neurological severity score and rotarod test. However, we could not demonstrate a reduction or compensation by contemporaneous flumazenil application. We did not observe effects on histological lesion volume by midazolam and/or flumazenil administration. This is inconsistent with our previous study, suggesting that posttraumatic application of the GABA-A receptor agonist propofol induced a proBDNF-p75NTR-dependent increase in brain damage, cell death, and impairment of motor function [8, 9].

Other experimental studies demonstrated that GABA-A modulators, such as benzodiazepines and propofol, may induce neurotoxicity in the young and developing brain [28–31]. These changes become apparent already after a short exposure to anesthetic agents [32]. Accumulating evidence suggests similarities between developmental and postinjury repair at molecular, cellular, and behavioral levels. These similarities could probably promote processes that, in turn, could render the brain sensitive to GABA-A-mediated neurotoxicity. This may be due to a shift of GABA-A receptor-mediated responses from hyperpolarization to developmental-like depolarization [33]. A study examining the effects of four sedation regimens, including isoflurane MAC 1.0, isoflurane MAC 1.67, midazolam alone, and midazolam combined with flumazenil, 2 h before inducing experimental TBI in rats demonstrated that sedation with isoflurane MAC 1.67 or midazolam increased the apoptotic cell count [34]. In contrast to these results, the neuroprotective effects of GABA-A receptor agonists have been described to reduce lesion size and improve functional outcomes in animal models of cerebral injury [35, 36]. Under our limited experimental conditions, midazolam was injected at a single time point after experimental trauma. The applied sedatives’ timing and dosage may be important for their ability and extend to induce neurotoxicity or neuroprotection. The same stimulus, depending on its relationship with brain insult, can be neuroprotective or cause neurodegeneration. Therefore, further experimental studies are required to clarify the time- and dose-dependent effects of benzodiazepine administration after TBI. We also evaluated the markers of posttraumatic neuroinflammation after a single-bolus injection of midazolam. Our results demonstrated that midazolam application slightly increased in the proinflammatory marker mRNA expression of *IL-1β* three days after TBI. This was reversed by flumazenil administration. An interaction between inflammatory mediators and excitatory signaling was demonstrated by blockading the glutamate NMDA receptor, thereby suppressing neuroinflammation after TBI [37, 38].

Currently, there are limited patient data on the impact of sedation or anesthesia on the outcome to facilitate surgery or intensive care therapy after acute cerebral injury or ischemia. A clinical study investigating cerebral biomarkers by microdialysis in patients suffering from severe TBI reported no difference in the metabolic profile between patients receiving propofol or midazolam sedation during the acute phase of TBI [39]. Consistent with our experimental data, a meta-analysis of randomized controlled trials comparing propofol and midazolam sedation demonstrated similar safety and efficacy profiles in patients suffering from severe TBI [40].

In summary, our data suggest that midazolam, in contrast to propofol, does show a delayed neurotoxic effect in injured brain tissue. The present study therefore adds important information for future clinical studies to identify sedative with a low neurotoxic profile for the treatment of brain injury patients.

## Conclusions

The present study investigated a single administration of midazolam in TBI using a highly standardized CCI model. We could not demonstrate an effect on histological brain damage by posttraumatic midazolam administration. Midazolam administration impaired functional recovery, and the administration of midazolam antagonist flumazenil could not counteract this effect. An increase in *IL-1β* mRNA expression levels by postinjury application of midazolam was reversible by flumazenil application. However, other inflammatory parameters remained unaffected. Therefore, our study results suggest a minor influence of single-bolus administration of midazolam on delayed pathophysiological mechanisms.

## Data Availability

All datasets generated and analyzed during this study are kept in the Dept of Anesthesiology, Medical Center of the Johannes Gutenberg-University and are available from the corresponding author upon reasonable request.

## Abbreviations

CCI: Controlled cortical impact
LORR: Loss of righting reflex
TBI: Traumatic brain injury
